# Underweight as a Risk Factor for Major Intra-abdominal Malignancy Surgeries

**DOI:** 10.7759/cureus.71835

**Published:** 2024-10-19

**Authors:** Jia Yin Lim, Yuhe Ke, Nian Chih Hwang

**Affiliations:** 1 Division of Anaesthesiology and Perioperative Medicine, Singapore General Hospital, Singapore, SGP

**Keywords:** abdominal surgery, body mass index, cancer, malignancy, underweight

## Abstract

Purpose

The relationship between body mass index (BMI) and postoperative outcomes for oncology surgeries among the Asian population remains controversial. A prevailing perception suggests that excess adiposity is associated with reduced cancer survival. However, several reports have shown that overweight or early obese states confer better survival outcomes. It is hypothesized that patients with better nutrition and surplus calories grant survival advantages to radical cancer treatment. The purpose of this study is to examine the relationship between BMI and postoperative 30-day and one-year mortality in the Asian population.

Methods

This retrospective review investigates the postoperative mortality, intensive care unit (ICU) admission, and length of hospital stay following major abdominal cancer surgeries within the Asian population. Patients were stratified into three groups based on their BMI: underweight (BMI < 18.5 kg/m^2^), normal weight (BMI 18.5-27.5 kg/m^2^), and obese (BMI > 27.5 kg/m^2^).

Results

A total of 646 patients were included in this study. At 30 days, the underweight group presents an 8% mortality compared to 1% and 3% mortality in the normal BMI and obese groups, respectively. At one year, the low BMI group presents a significant increase in mortality of 53% as opposed to the normal BMI and obese group with mortality rates of 14% and 12%, respectively (p < 0.001). There is a significant increase in the ICU admission rate in the underweight group (n = 13, 25%) compared to the normal BMI and obese groups (n = 26, 6%; n = 8, 6% p < 0.001). Similarly, the group with lower BMI was observed to require a longer hospital stay postoperatively (median (IQR) 11.0 (4.0-24.0)) compared to the normal (median (IQR) 4.0 (3.0-9.0)) and obese (median (IQR) 4.5 (3.0-8.0)) patients.

Conclusion

Concordant results were observed in the underweight patient group with increased one-year mortality, ICU admission rate, and hospital stay postoperatively. Low BMI presents as an independent risk factor for major radical surgeries.

## Introduction

Obesity is a significant global public health challenge. The World Cancer Research Fund (WCRF) reported strong evidence to suggest that body adiposity is associated with increased incidence of at least 13 cancer types including esophageal, pancreas, liver, colorectal, postmenopausal breast, and renal malignancies [1]. Thus, maintaining a healthy weight is one of the key modifiable cancer prevention recommendations [2].

In recent years, the association between body mass index (BMI) and postoperative outcomes of cancer surgery has been extensively studied [3]. An elevated BMI was regarded as a poor prognostic factor for many surgical interventions in cancer management [4-6]. In contrast to the expected worsened surgical outcome associated with obesity, several reports advocated the phenomenon of the “obesity paradox” when overweight and early obese states are associated with improved survival outcomes [7-9]. It is hypothesized that excessive adiposity and surplus calories grant survival advantages to radical cancer treatment [10].

Reports on the relationship between BMI and postoperative outcomes for oncology surgeries among the Asian population remain lacking and controversial. The prevalence of obesity in the Asian population differs from the Western population [11]. It was also established that Asians of the same BMI, sex, and age as Caucasians have a greater proportion of body fat and are hence associated with an increased risk of comorbidities and cardiovascular diseases [12,13]. Therefore, whether the phenomenon of the “obesity paradox” reflected in the Western population can be generalized in the Asian population remains unknown. This report aims to examine the association between BMI and the postoperative 30-day and one-year mortality of major intra-abdominal oncology surgeries in the Asian population.

## Materials and methods

This is a retrospective review of patients with intra-abdominal cancers undergoing major intra-abdominal surgeries in Singapore General Hospital, a tertiary hospital in Singapore, between 2013 and 2017. Ethical approval for this study was obtained from the SingHealth Centralised Institutional Review Board (reference number 2015/2468). The written consent requirement was waived for the purpose of this study.

The electronic health records of 14,072 patients who underwent elective and emergency intra-abdominal surgeries were reviewed. The majority of the patients came in for non-cancer operations including caesarean section delivery. Patients who underwent moderate and high-risk intra-abdominal surgeries for malignancy were assessed for eligibility. We have excluded patients under the age of 18 years old. Patients with missing data in crucial fields such as BMI, type of surgery, and risk of surgery were excluded. The patient data were retrieved up to one year from the operative date where the one-year mortality and ICU and hospital readmission rates were recorded.

The electronic medical records for the patients were obtained from the institutional clinical information system (Sunrise Clinical Manager (SCM), Allscripts, Illinois, USA). These records are deposited in our enterprise data repository and analytics system (SingHealth-IHiS Electronic Health Intelligence System). All the preoperative data were extracted from the preoperative anesthesia evaluation chart during the patient's visit to the preoperative anesthesia clinic. Preoperative anesthesia evaluation of the patient is usually performed two weeks prior to the date of operation. To ensure near-complete follow-up, mortality records were matched with the data from the National Registry of Disease Office, Singapore. Survival days were determined from the date of hospital admission to death. The reason for demise was not included. The study is reported in accordance with the Strengthening the Reporting of Cohort Studies in Surgery (STROCSS) guideline 2019.

Patients were stratified into three groups based on BMI: underweight (BMI <18.5 kg/m^2^), normal weight (BMI 18.5-27.5 kg/m^2^), and obese (BMI >27.5 kg/m^2^). This BMI classification was applied based on the scientific evidence review by the WHO expert panel from various Asian countries including Singapore [14].

Statistical analysis

The missing data for all variables account for <2%. These missing values were substituted with the medians in continuous variables and the mode in categorical variables. Variables with >2% missing data were removed from the analysis as they were non-essential for the intention of this study.

Patient demographics and clinical characteristics were stratified by BMI classes. The mean and standard deviation (SD) were presented for continuous variables, and the mean differences between the groups were tested using the Mann-Whitney U test. For categorical variables, a comparison between the groups was conducted using Fisher’s exact test. A multivariable logistic regression model was used to determine the independent variables that correlated significantly with mortality and length of ICU stay. The multivariable regression models included factors with a P-value of < 0.1 from the univariable analysis. The effect size was presented as odds ratio (OR) and 95% confidence interval (CI).

Bonferroni-adjusted P-value for the multivariable models was kept conservatively at P < 0.001 as more than two comparisons were made (BMI categories, American Society of Anesthesiologists physical (ASA) status) on multiple occasions.

All statistical computing, visualizations, and analyses were conducted using the R environment version 1.2.1335 (The R Foundation for Statistical Computing, Vienna, Austria). Violin plot, bar plot, and Kaplan-Meier curve were plotted and quantified with the R library package [15].

The primary outcome was the association of different BMI groups with 30-day and one-year all-cause mortality. The secondary outcome was the rate of postoperative ICU admission and length of hospital stay.

## Results

**Figure 1:**
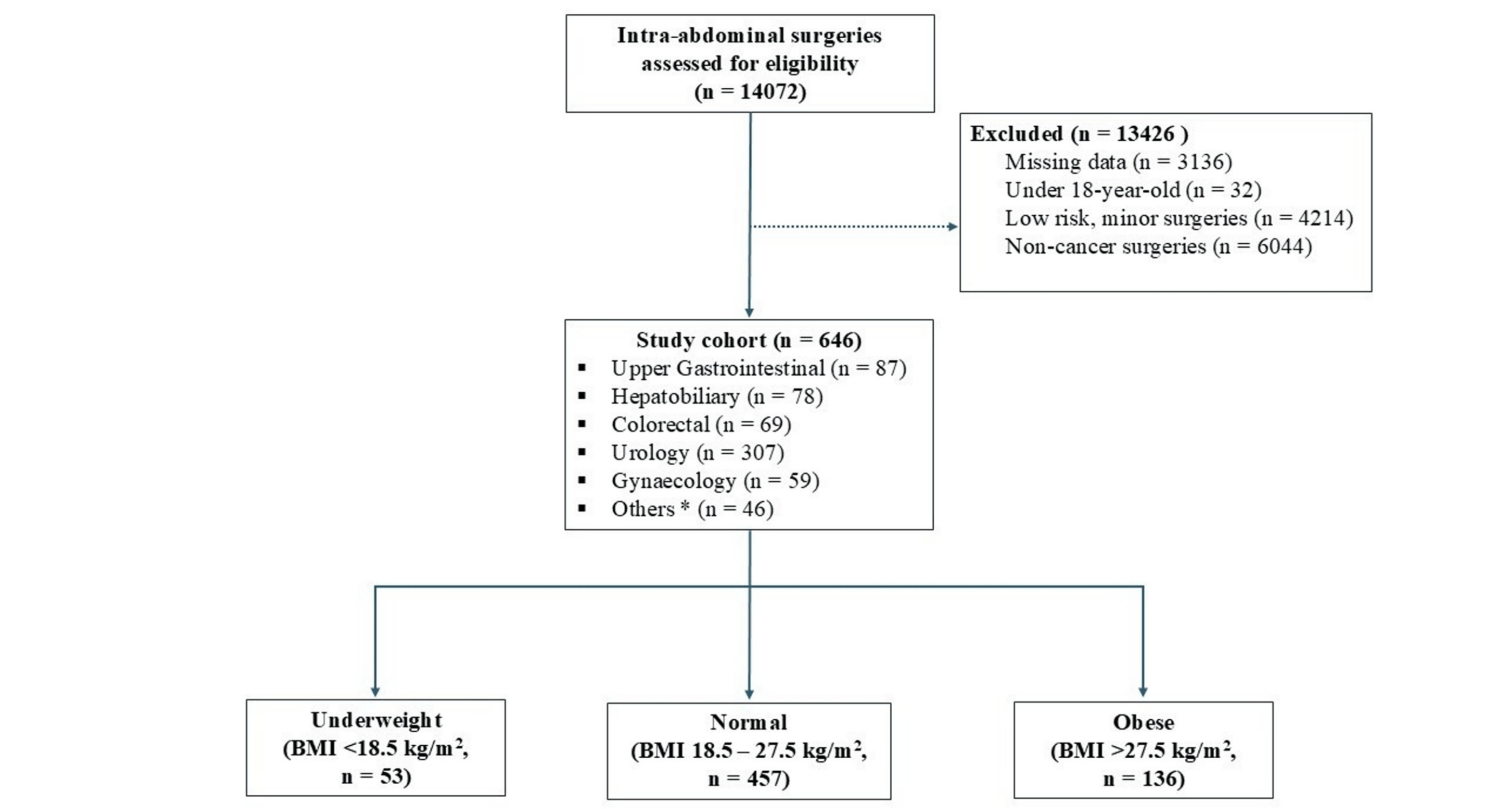
Flowchart of the study cohort * Peritoneal/retroperitoneal disease, liposarcoma, lymphoma, malignant neurofibromatosis, sacral chordoma, intra-abdominal tumor of unknown origin

A total of 14,072 patients underwent intra-abdominal surgeries over a five-year period (2013-2017) in our institution. Our final evaluation was conducted with 646 patient observations (Figure 1). These patients were categorized into their respective BMI classifications of underweight (BMI <18.5 kg/m^2^, n = 53 (8%)), normal weight (BMI 18.5-27.5 kg/m^2^, n = 457 (71%)), obese (BMI >27.5 kg/m^2^, n = 136 (21%)). The baseline characteristics of these patients are presented in Table 1.

**Table 1:** Patient characteristics stratified by different BMI groups. ^1^ n (%); Mean (SD); Median (IQR). ^2^ The statistical tests used include the chi-square test for categorical variables, the Kruskal-Wallis rank sum test for continuous variables, and Fisher's exact test for small group sizes. Statistical significance was considered at p < 0.05. ^3^ American Society of Anesthesiologists (ASA). ^4^ Angiotensin II receptor blocker (ARB), angiotensin-converting enzyme inhibitor (ACEi).

Variable	Overall, N = 646^1^	Underweight, N = 53^1^	Normal, N = 457^1^	Obese, N = 136^1^	P-value^2^
Age (years)					0.2
18-49	79 (12%)	11 (21%)	48 (10%)	20 (15%)	
50-64	252 (39%)	21 (40%)	178 (39%)	53 (39%)	
64-80	315 (49%)	21 (40%)	231 (51%)	63 (46%)	
BMI	24.03 (4.38)	16.66 (1.52)	23.04 (2.30)	30.22 (3.09)	<0.001
Gender					<0.001
Female	206 (32%)	29 (55%)	126 (28%)	51 (38%)	
Male	440 (68%)	24 (45%)	331 (72%)	85 (62%)	
Race					<0.001
Chinese	525 (81%)	46 (87%)	388 (85%)	91 (67%)	
Indian	25 (3.9%)	0 (0%)	14 (3.1%)	11 (8.1%)	
Malay	24 (3.7%)	3 (5.7%)	10 (2.2%)	11 (8.1%)	
Others	72 (11%)	4 (7.5%)	45 (9.8%)	23 (17%)	
Repeat surgery	29 (4.5%)	5 (9.4%)	19 (4.2%)	5 (3.7%)	0.2
Operation priority					<0.001
Elective	592 (92%)	40 (75%)	424 (93%)	128 (94%)	
Emergency	54 (8.4%)	13 (25%)	33 (7.2%)	8 (5.9%)	
Surgery type					<0.001
Hepatobiliary	78 (12%)	8 (15%)	56 (12%)	14 (10%)	
Colorectal	69 (11%)	10 (19%)	48 (11%)	11 (8.1%)	
Upper gastrointestinal	87 (14%)	13 (25%)	56 (12%)	18 (13%)	
Urology	307 (48%)	9 (17%)	229 (50%)	69 (51%)	
Gynecology	59 (9.1%)	5 (9.4%)	37 (8.1%)	17 (12%)	
Others	46 (7.1%)	8 (15%)	31 (6.8%)	7 (5.1%)	
ASA status ^3^					<0.001
1	55 (8.5%)	6 (11%)	40 (8.8%)	9 (6.6%)	
2	509 (79%)	33 (62%)	366 (80%)	110 (81%)	
3	79 (12%)	13 (25%)	49 (11%)	17 (12%)	
4	3 (0.5%)	1 (1.9%)	2 (0.4%)	0 (0%)	
Surgical risk					0.009
Low risk	63 (9.8%)	9 (17%)	41 (9.0%)	13 (9.6%)	
Intermediate risk	545 (84%)	36 (68%)	390 (85%)	119 (88%)	
High risk	38 (5.9%)	8 (15%)	26 (5.7%)	4 (2.9%)	
Hemoglobin (Hb) level					
Hb < 13	164 (25%)	30 (57%)	108 (24%)	26 (19%)	0.4
Hb < 9	15 (2.3%)	2 (3.8%)	7 (1.5%)	6 (4.4%)	0.2
Hb < 7	2 (0.3%)	1 (1.9%)	0 (0%)	1 (0.7%)	0.10
Chronic medications					
Statin	189 (29%)	7 (13%)	133 (29%)	49 (36%)	0.008
Beta blocker	108 (17%)	3 (5.7%)	74 (16%)	31 (23%)	0.015
ARB/ACEi ^4^	116 (18%)	5 (9.4%)	74 (16%)	37 (27%)	0.003
Calcium channel blockers	153 (24%)	5 (9.4%)	97 (21%)	51 (38%)	<0.001
Antidiabetic	88 (14%)	8 (15%)	55 (12%)	25 (18%)	0.2
Insulin	37 (5.7%)	6 (11%)	20 (4.4%)	11 (8.1%)	0.042
Aspirin	52 (8.0%)	1 (1.9%)	37 (8.1%)	14 (10%)	0.2
Clopidogrel	22 (3.4%)	3 (5.7%)	17 (3.7%)	2 (1.5%)	0.3
Warfarin	2 (0.3%)	0 (0%)	1 (0.2%)	1 (0.7%)	0.5
Enoxaparin	14 (2.2%)	4 (7.5%)	6 (1.3%)	4 (2.9%)	0.014
Other anticoagulant	25 (3.9%)	5 (9.4%)	13 (2.8%)	7 (5.1%)	0.039
Comorbidities					
Ischemic heart disease	23 (3.6%)	0 (0%)	19 (4.2%)	4 (2.9%)	0.4
Cerebrovascular events	6 (0.9%)	3 (5.7%)	3 (0.7%)	0 (0%)	0.017
Chronic kidney disease stage ≥ 4 or end-stage renal failure	5 (0.8%)	1 (1.9%)	4 (0.9%)	0 (0%)	0.3
Hypertension	148 (23%)	6 (11%)	103 (23%)	39 (29%)	0.036
Hyperlipidemia	120 (19%)	3 (5.7%)	87 (19%)	30 (22%)	0.030
Diabetes mellitus	68 (11%)	3 (5.7%)	43 (9.4%)	22 (16%)	0.038
Atrial Fibrillation	11 (1.7%)	0 (0%)	8 (1.8%)	3 (2.2%)	0.8
Metabolic equivalents (MET)					0.007
MET 1-4	25 (4.3%)	7 (16%)	11 (2.7%)	7 (5.4%)	
MET 4-10	539 (92%)	37 (82%)	386 (93%)	116 (90%)	
MET >10)	24 (4.1%)	1 (2.2%)	17 (4.1%)	6 (4.7%)	
Length of hospital stay (days)					<0.001
Median (IQR)	5.0 (3.0, 9.0)	11.0 (4.0, 24.0)	4.0 (3.0, 9.0)	4.5 (3.0, 8.0)	
Range	0.0, 172.0	0.0, 172	0.0, 124.0	0.0, 135.0	

Primary outcome (30-day and one-year mortality)

The rate of all-cause mortality was 1.7% (n = 11) and 16.7% (n = 108) at 30 days and one year, respectively. The results of the univariate and multivariate analyses for 30-day and one-year mortality are presented in Table 2. A statistically significant association was observed between 30-day mortality and BMI, gender, and ASA status. At 30 days, the underweight group presents with an 8% (n = 4) mortality compared to 1% (n =3) and 3% (n = 4) mortality in the normal BMI and obese groups respectively. Nonetheless, the multivariate analysis at 30 days revealed a non-statistically significant increase in mortality in both the underweight and obese groups (OR 1.08, 95% CI, 0.984-1.189; p = 0.10; OR 1.11, 95% CI, 1.004-1.219; p = 0.043) respectively.

**Table 2:** Univariate and multivariate analyses of outcome and BMI groups ^1 ^Data are represented as n (%). ^2 ^Logistic regression was used to calculate the odds ratios (OR) and p-values. Statistical significance was considered at p < 0.001 (Bonferroni adjusted). ^3^ CI = confidence interval. Covariate adjusted for gender, race, ASA status, anemia (Hb < 13 g/dL), metabolic equivalents, hypertension, and emergency operation.

			Univariate				Adjusted		
	No^1^	Yes^1^	Odds ratio^2^	95% CI^3^	Wald chi-square	p- value^2^	Odds ratio^2^	95% CI^3^	p-value^2^
30-day mortality									
Underweight (<18.5 kg/m^2^)	49 (92%)	4 (8%)	1.07	1.03, 1.11	12.59	<0.001	1.08	0.984, 1.189	0.10
Normal (18.5 - 27.5 kg/m^2^)	454 (99%)	3 (1%)	-	-			-	-	-
Obese (>27.5 kg/m^2^)	132 (97%)	4 (3%)	1.02	0.998, 1.137	3.33	0.068	1.11	1.004, 1.219	0.043
One-year mortality									
Underweight (<18.5 kg/m^2^)	25 (47%)	28 (53%)	1.48	1.335, 1.643	27.15	<0.001	1.35	1.11, 1.63	0.001
Normal (18.5-27.5 kg/m^2^)	394 (86%)	63 (14%)	-	-		-	-	-	-
Obese (>27.5 kg/m^2^)	119 (88%)	17 (12%)	0.99	0.922, 1.058	0.15	0.7	1.00	0.826, 1.22	>0.9
ICU admission									
Underweight (<18.5 kg/m^2^)	40 (75%)	13 (25%)	1.21	1.123, 1.298	19.88	<0.001	1.31	1.12, 1.51	<0.001
Normal (18.5-27.5 kg/m^2^)	431 (94%)	26 (6%)	-	-		-	-	-	-
Obese (>27. 5kg/m^2^)	128 (94%)	8 (6%)	1.00	0.954, 1.052	0.12	>0.9	0.99	0.204, 1.16	>0.9

All preselected variables other than hypertension showed a statistically significant effect on the overall mortality at one year. At one-year follow-up, the low BMI group presents a significant increase in mortality of 53% (n = 28) as opposed to the normal BMI and obese group with a mortality rate of 14% (n = 63) and 12% (n = 17), respectively (p < 0.001). Following the multivariate analysis, the low BMI group remained an independent prognostic factor for one-year mortality (OR 1.35, 95% CI, 1.11-1.63; p = 0.001).

**Figure 2:**
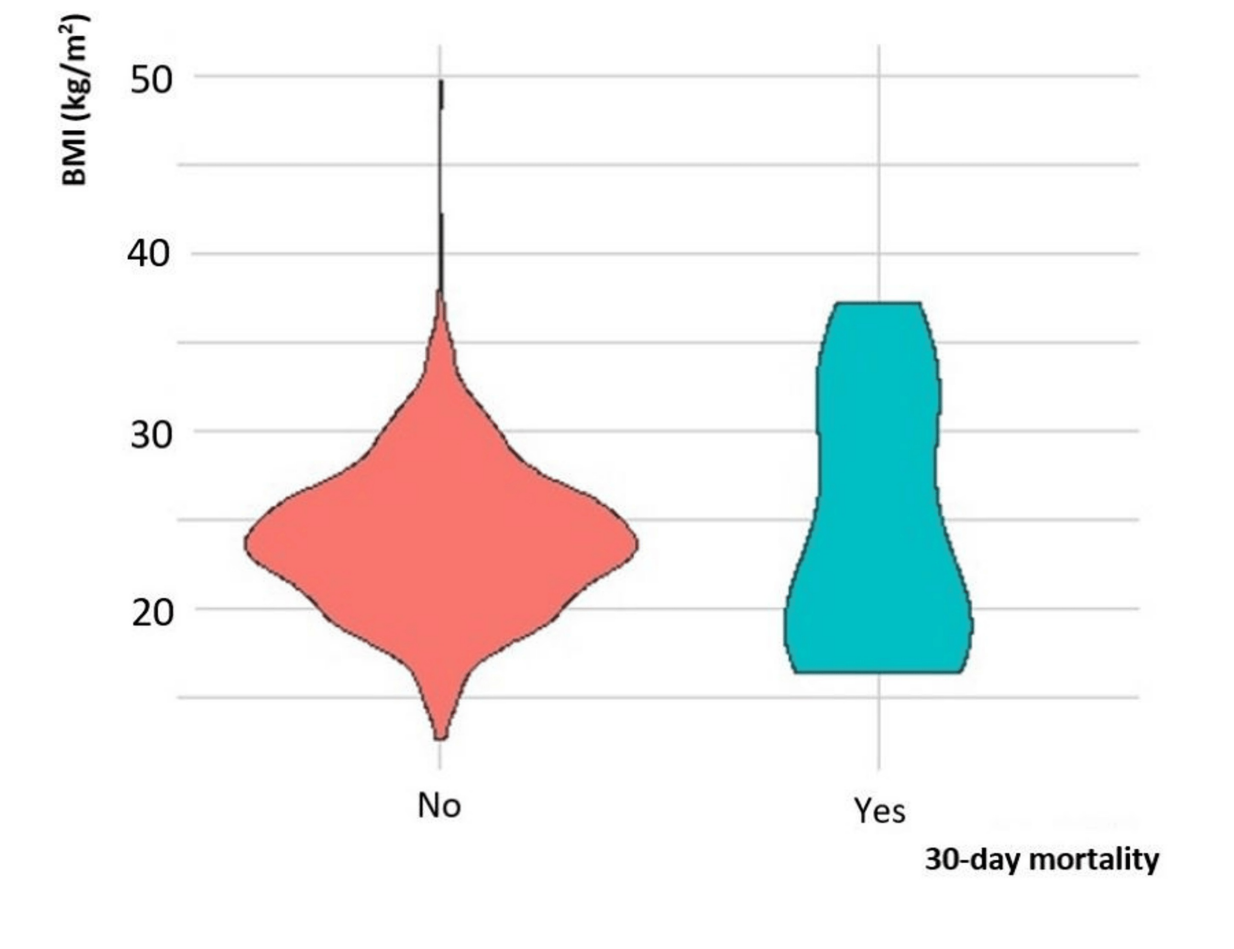
Violin plot of association of BMI (kg/m2) and 30-day mortality.

**Figure 3:**
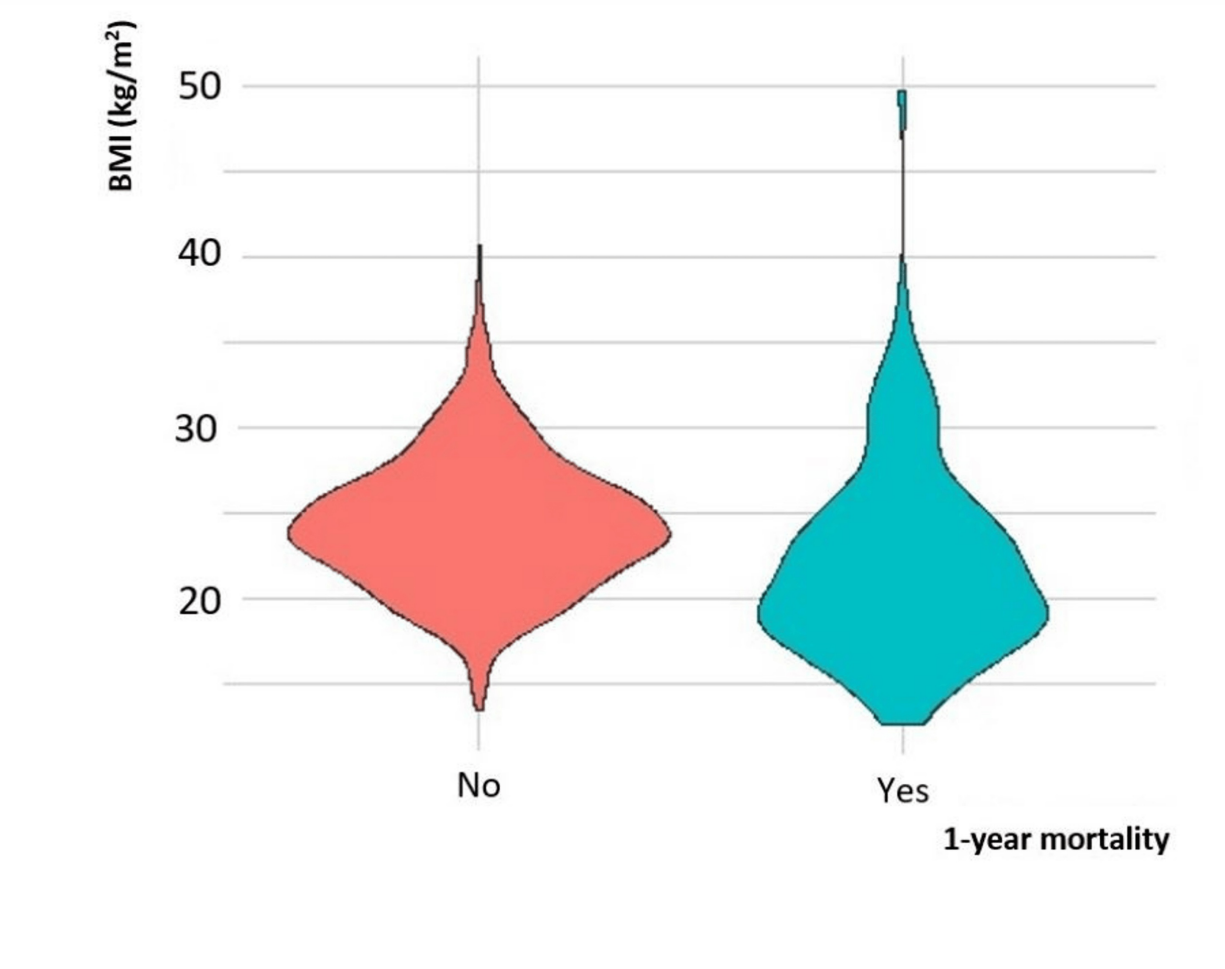
Violin plot of association of BMI (kg/m2) and one-year mortality.

**Figure 4:**
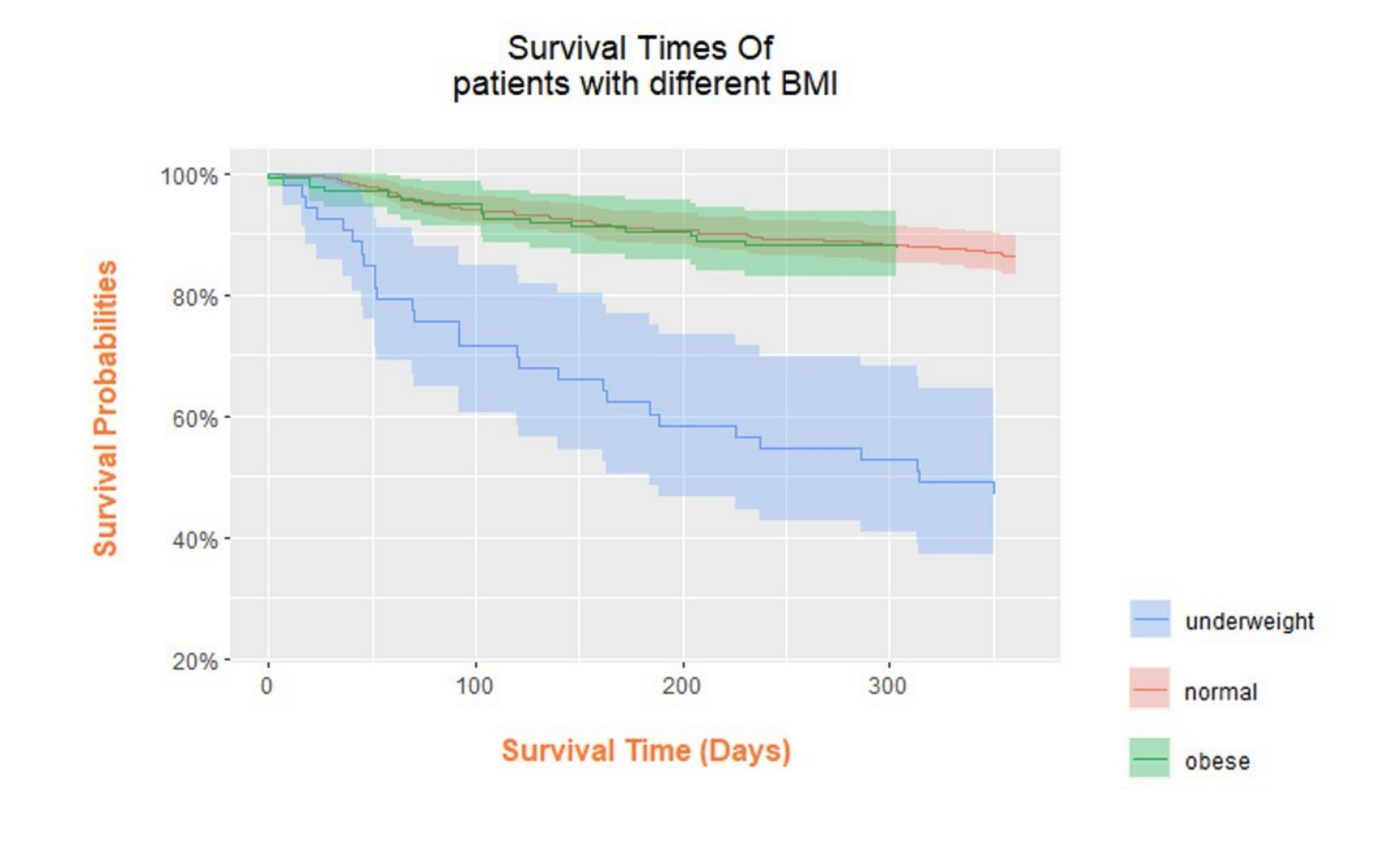
Kaplan-Meier curve of the one-year postoperative survival outcome and different BMI categories.

As described earlier, we observed a marginal increase in the 30-day all-cause mortality in the underweight group compared to the normal BMI and obese groups. A biphasic distribution of mortality was observed at 30 days postoperatively, greatest with the underweight and obese groups (Figure 2). In one year, there was a significant rise in mortality incidence, most of which underwent hepatobiliary (27.8%), colorectal (25%), or upper gastrointestinal (19%) surgeries (Figure 3). The Kaplan-Meier curve also presents the long-term survival outcome of patients in their various BMI subgroups (Figure 4). Patients with BMI < 18.5 kg/m^2^ had the highest postoperative mortality rate, while the high-BMI group presented the best one-year postoperative survival outcome.

Secondary outcome (ICU admission and length of hospital stay)

A total of 47 (7.3%) patients required ICU admission during their postoperative hospital stay. A strong association between ICU admission with BMI and ASA status was observed. There is a significant increase in the ICU admission rate in the underweight group (n = 13, 25%) compared to the normal BMI and obese groups (n = 26, 6%; n = 8, 6%, p ≤ 0.001), respectively. Upper gastrointestinal cancer surgeries (n = 21, 44.6%) appear to contribute to most of the postoperative ICU admissions. This is followed by colorectal (n = 7, 14.9%), hepatobiliary (n = 6, 12.8%), urology (n = 4, 8.5%), gynecology (n = 2, 4.3%), and other (n = 7, 14.9%) cancer surgeries.

**Figure 5:**
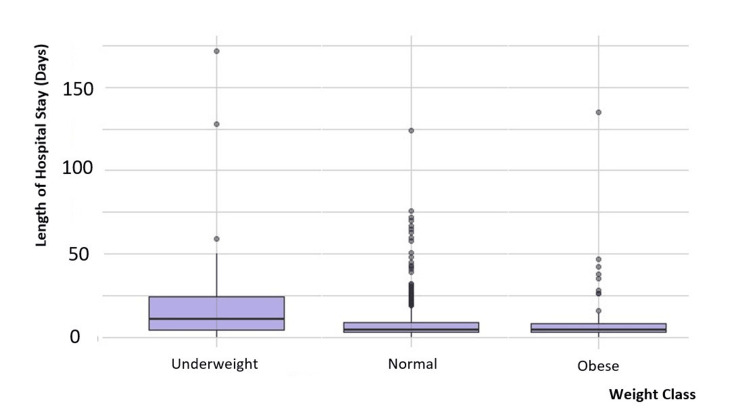
Postoperative length of hospital stays with different BMI categories.

Similarly, the group with lower BMI was observed to require a longer hospital stay postoperatively (median (IQR) 11.0 (4.0-24.0)) compared to the normal (median (IQR) 4.0 (3.0-9.0)) and obese (median (IQR) 4.5 (3.0-8.0)) patients (Figure 5 and Table 1).

Concordant results were observed in the underweight patient group with increased one-year mortality, ICU admission rate, and hospital stay postoperatively.

## Discussion

Many studies in the past have reported an elevated BMI to reflect less favorable surgical outcomes, including longer operative time, higher risk of postoperative complications, and prolonged hospital stay [16]. In our study, low BMI was recognized as an independent prognostic factor for mortality, hospital stay, and ICU admission. This is congruent with the findings of a recent multicenter study in Taiwan by Hung et al., which reported a 3.8-fold increase in all-cause mortality in patients with BMI ≤17 kg/m^2^ [17]. On the contrary, Yamamoto et al. and Son et al. reported either no difference in the postoperative outcome or increased postoperative complication for those with a high BMI [18,19]. Notably, there was variation in the BMI cut-off applied by each study.

In line with the conflicting surgical outcomes associated with different BMI groups, our analysis expands to explore the demographic and clinical variations across BMI categories. Specifically, the underweight group included a higher proportion of younger individuals (18-49 years), potentially reflecting a younger, malnourished population with advanced oncological disease. The race distribution revealed a larger proportion of Chinese patients, which reflects the ethnic makeup of the local population, where Chinese individuals form the majority. Obesity, however, was more prevalent among Malay and Indian patients, consistent with regional trends in BMI and metabolic disorders. Furthermore, chronic medication use, particularly statins, beta-blockers, and calcium channel blockers, was significantly higher in the obese group, aligning with the increased cardiovascular comorbidities in this population. These demographic and clinical differences may contribute to the variability in surgical outcomes and underscore the need for tailored perioperative management strategies.

Contrary to the mortality distribution at one year, a biphasic distribution of 30-day mortality was observed, the greatest among the underweight and obese groups. The increase in short-term mortality in the obese group is possibly a reflection of increased immediate postoperative complications such as pulmonary complications and anastomotic leak [20,21]. At one year, the mortality difference in our study became more apparent with a 3.8-fold to 4.4-fold increased risk in all-cause mortality for patients with BMI <18.5 kg/m^2^ when compared with the normal BMI and overweight groups. Obesity presents the best survival probability compared to the normal BMI and underweight groups. These observations are in line with the phenomenon of the “obesity paradox” observed and the Asian BMI cut-offs allow for congruent findings.

It is observed that all the underweight groups with high surgical risk scores underwent upper gastrointestinal or pancreatic surgeries. Hepatobiliary and upper gastrointestinal cancers are among those with the poorest survival rates [22]. As expected, we observe a high ICU admission (75%) and one-year mortality rate (50%) in this group of patients. The late presentation and diagnosis of such cancers may be another plausible explanation for the high ICU admission and mortality. According to the Singapore Cancer Registry Report 2019, over 60% of stomach cancers and 74.5% of pancreatic cancers were diagnosed with stage III or IV of the disease [22]. For the reasons mentioned, upper gastrointestinal cancer surgeries account for the majority of the ICU admissions (44.7%), while hepatobiliary cancer surgeries presented the highest one-year mortality (27.8%) in our study.

Radical cancer surgery is an important curative modality for many solid tumors [23]. Such intensive treatment is associated with high morbidity and mortality, especially for those with extensive tumor disease, poor premorbid status, or frail [24,25]. Many cancer patients are burdened by protein-energy malnutrition because of reduced caloric or protein intake and elevated resting energy expenditure associated with malignancy [26]. This leads to weight loss and lower mean plasma albumin concentrations, both of which are known risk factors for undesirable surgical outcomes [27,28]. As the metabolic regulation and immunologic response are heavily integrated, the malnourished state causes impaired immunological response and compromised antitumor response [29]. Conversely, a mild obese state with metabolic and nutrient surplus triggers a low-grade, chronic inflammatory state, which is primed to mount the appropriate immunological and inflammatory response to surgical stressors [30].

Nutritional deficiency is known to be a common cause of anemia [31]. As such, one would expect patients with poor nutritional reserves to have a higher incidence of anemia. However, this was not observed in our study population. Hence, the increased mortality in the malnourished or underweight group was not attributed to anemia.

Over the past years, cancer prehabilitation has been widely discussed and investigated. It is the process in the continuum of cancer care that occurs between the time of cancer diagnosis and cancer treatment [32]. Preoperative nutritional support as one of the main components in cancer prehabilitation, can improve post-surgical outcomes with fewer infective or non-infective complications, shorten hospital length of stay, and reduce short-term mortality [33,34]. Based on the latest European Society for Clinical Nutrition and Metabolism (ESPEN) recommendation, preoperative nutritional intervention for patients with malnutrition should be undertaken for a minimum of 10-14 days [35]. Enteral nutrition remains the preferred and physiological feeding method [36]. Despite the lack of evidence for parental nutrition, it is recommended when the enteral route is prohibited. With our study confirming poor perioperative outcomes in the underweight group, a multidisciplinary approach, and cancer prehabilitation with time-sensitive delay should be considered before planning for acute cancer treatment and surgical resection.

Limitations

This observational cohort study investigated the impact of BMI on 30-day and one-year mortality. This study is reflective of our local Asian population where the Asian-BMI criteria were utilized for analysis. However, there are some limitations as follows. Selection bias may exist in this retrospective review where the morbidly obese patients with poor premorbid and performance status were deemed unfit for major radical surgery. As a result, the high BMI patients included in this study were associated with better physical and performance status compared to the low BMI group. Hence, underestimating the true mortality risk in obese patients.

Although the cohort looks at oncological patients, traditional outcomes such as a five-year disease-free survival period are not reported as the focus of the data is on the perioperative period and mortality at 30 days and one year. A longer duration of follow-up would be beneficial to ascertain the impact of weight difference on long-term disease-free survival in patients after surgery. Other oncological-related parameters such as state of disease and metastasis were not included due to data input limitations as the data was extracted from perioperative anesthesia notes.

The heterogeneous distribution of weight classes across different surgery types, coupled with variation in chronic medications and comorbidities may introduce biases, which make comparison between groups challenging. Disparities in the weight distribution could stem from differences in the timing of presentation. For instance, urological cancers typically present earlier due to robust screening tests compared to other cancers. Our study results may also be confounded by the fact that the underweight group has a higher proportion of patients with lower metabolic equivalents (METs) and American Society of Anesthesiology (ASA) physical health status 3 or 4. It is thought that the reduced BMI represented the underlying advanced tumor burden and malnourished state with weight loss and muscle wasting, all of which confer a poor surgical outcome and diminished reserve for oncological treatment.

Lastly, patients undergoing major surgeries experience significant physiological stress, leading to a hypercatabolic state and high energy expenditure [37]. Hence, malnutrition in the critically ill is associated with critical illness myopathy, neuropathy, and poor outcomes including increased risk of infection, prolonged mechanical ventilation, and mortality [38]. However, ventilation days and postoperative complications such as infection were not included in our dataset. Therefore, this study was unable to demonstrate the association between preoperative disease state and surgical complications or perioperative morbidity.

## Conclusions

In patients undergoing major intra-abdominal malignant surgeries, underweight cancer patients with BMI <18.5 kg/m^2^ have significantly poorer outcomes with 8% and 53% mortality at 30 days and one year, respectively. The underweight patient group also presents a significantly higher ICU admission rate and longer length of hospital stay. In conclusion, low BMI is an independent risk factor for major radical surgeries.
